# Effect of Phenobarbital on Chloramphonicol-Induced Toxicity in Rat Liver and Small Intestine

**Published:** 2013-12

**Authors:** Massumeh Ahmadizadeh, Masood Esmailpoor, Zahra Goodarzi

**Affiliations:** 1 Physiology, Toxicology and Social Determinants of Health Research Centers of Ahvaz Jundishapur University of Medical Sciences, Ahvaz, Iran; 2 Occupational Health Department of Public Health School, , Ahvaz Jundishapur University of Medical Sciences, Ahvaz, Iran

**Keywords:** Chloramphenicol, Liver, Phenobarbital, Small intestine

## Abstract

***Objective(s): ***The aim of the present study is to determine the effect of Chloramphenicol (CAP) on rat liver and small intestine. Effect of phenobarbital (PB) on CAP toxicity was also investigated.

***Materials and Methods:*** Rats were received CAP at doses of 0, 200, 400 and 600 mg/kg. Another group was pretreated with 80 mg/kg PB 30 min prior to administration of various doses of CAP. The experiment was repeated for seven consecutive days. Blood was collected for determination of serum aspartate aminotransferase (AST) alanine aminotransferase (ALT). The liver and small intestine tissues were processed for light microscopy.

***Results:*** CAP induced a dose dependent elevation of AST and ALT and produced injury in the liver and small intestine when compared to control animals. PB markedly decreased AST and ALT levels and protected liver and small intestine against CAP-induced toxicity.

***Conclusion*** *: *This study suggested rat liver and small intestine have potential to bioactivate CAP.

## Introduction

Chloramphenicol (CAP) is a broad-spectrum antibiotic affecting both Gram- positive and Gram- negative organisms. It is used in many parts of the world for the treatment of life-treating infections including typhoid fever and meningitis (1-3). Negative side effects of CAP are reported not only due to enteral or parental rout, but also from ocular administration (4-6). Adverse effects of CAP on liver tissue has been reported by several investigators (6-8). Effiong* et al* (2010) reported CAP-produced liver damage in rats (7). 

Saba *et al* (2000) found that prolonged administration of CAP could cause liver and kidney damage (8). However, only very limited studies reported effects of this agent on intestinal epithelial cells. Cybulska *et al* (1988) reported that therapeutic dose of CAP caused structural alterations in microvilli of the epithelial cells of the small intestine in hen (9).

Xenobiotic metabolizing enzyme system of the small intestinal is principally an initial source for metabolizing the ingested xenobiotics which results in facilitating the excretion. It has been found that CAP leads to a dose-dependent inhibition of the activity of the xenobiotic enzyme system.

A large body of evidence indicate that CAP is metabolized by hepatic cytochrome p450 (6, 10). Toxic effects of CAP on rat liver was studied by several investigators (6-8). The presence of cytochrome p450 in rat small intestine was reported (11-14). 

Phenobarbital (PB) is a sedative drug which is also used as a cytochrome p450 inducer. It has been shown that PB enhances cytochrome p450 activities in the liver and reduces hepatotoxicity of CAP (15).

Since studying the effect of CAP on GI system in experimental animals may be of use for better understanding of its clinical picture, the present study was undertaken to determine the effect of CAP on small intestine epithelial cells. In order to make a comparison, the effect of CAP on the liver was also investigated and to clarify the mechanism of CAP-induced toxicity, the effect of PB on CAP produced adverse effect was also investigated.

**Table 1:** Effect of phenobarbital (PB) on chloramphenicol induced alterations of aspartate aminotransferase (AST) and a alanine aminotransferase (ALT)

Parameters	Treatment	Doses (mg/kg)			
		0	200	400	600
AST	CAP	169.3±4.2	213.6±4.12*	267.4±8.2*	341.2±18.3*
	PB+CAP	165.1±4.2	189.2 ±3.2	212.3± 3.2	244.4 ±8.2
ALT	CAP	92.2± 3.5	142.2 ±3.2*	160.3± 6.2*	180.1 ±8.2*
	PB+CAP	89.1± 4.2	112.3 ±2.15	120.6 ±4.5	134.2 ±4.2

AST: aspartate aminotransferase; ALT: alanine aminotransferase; CAP: Chloramphenicol; PB: Phenobarbital

* Significantly different from rats pretreated with PB and were given the same dose of CAP; (*P*≤0.05)

## Materials and Methods

Adult male Sprague –Dowley rats (150-200 g weight) were housed in groups of 3 in clear polypropylene cages in a light cycle (12 hr light and 12 hr dark) in a temperature-controlled room. The animal was allowed having food and tap water .Rats were treated with CAP at doses of 200,400 and 600 mg/kg. Control animals were given vehicle only. At the same time, another group of animals were pretreated with 80 mg/kg PB or saline (vehicle) 30 min prior to administration of various doses of CAP. The experiment was repeated for seven consecutive days. 24 hr after the last administration, all animals were sacrifised. Blood was collected for determination of serum aspartate aminotransferase (AST) and alanine aminotransferase (ALT). The liver and small intestine tissues were removed, fixed and processed for light microscopy, using hematoxylin-eosin (*H & E*) staining technique. Five histological sections, each at least 15 µm apart, were taken from every tissue block and stained with H&E. The criteria for cell injury included nuclear dilation, loss of staining capacity and obvious cellular swelling. Ten animals were used in each treating group. The protocol was approved by the Ethics committee of the Ahvaz Jundishapur University of Medical Sciences.

Biochemical data were expressed as mean± standard error. The results of biochemical parameters were analyzed by analysis of variance, completely randomized design, and treatment differences were identified by the method of Newman-Keuls.*P*<0 .05 was used as the criterion for significance. 

## Results

CAP induced dose-dependent elevation of AST and ALT when compared to control animals. PB significantly decreased ALT and AST levels in CAP-treated rats when compared to those which received the same dose of CAP only (Table1).

**Figure 1:**
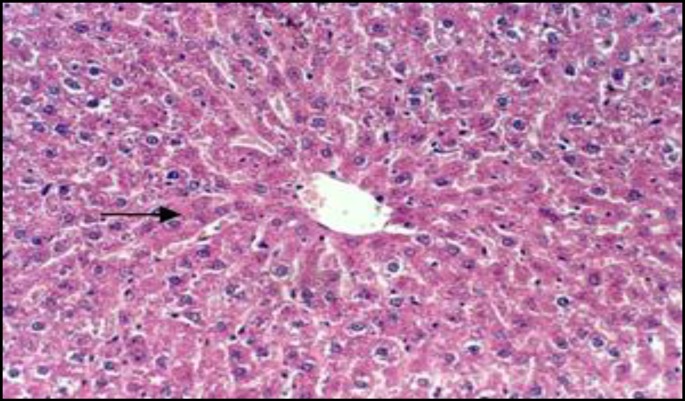
Light micrograph of rat liver treated with vehicle only (control). Hepatocytes (arrow) are intact. H&E* x* 200

**Figure 2:**
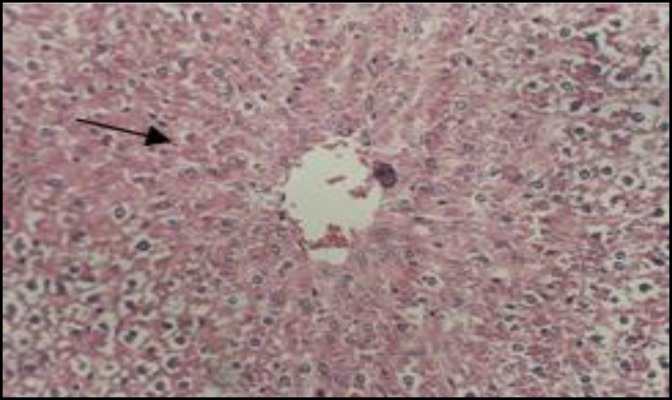
Light micrograph of rat liver treated with 600 mg/kg CAP. Shows marked damages in hepatocytes (arrow).H&E 200x

**Figure 3:**
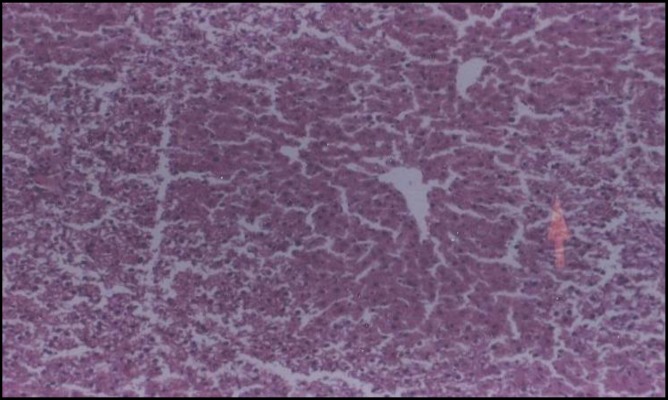
Light micrograph of rat liver pretreated with 80 mg/kg PB 30 min prior to administration of 600mg/kg CAP. Hepatocytes (arrow) appeared to be normal. H&E 200x

**Figure 4:**
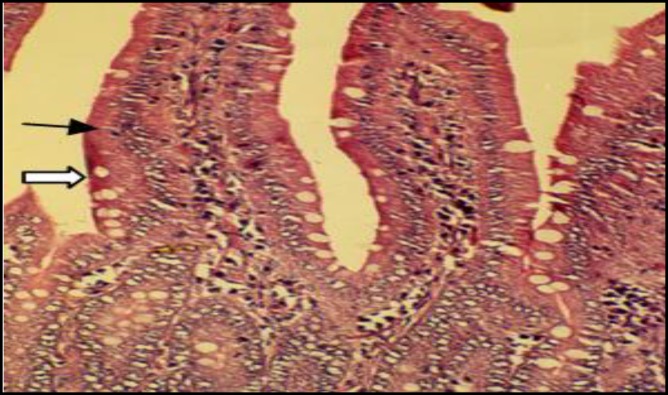
Light micrograph of rat small intestine treated with vehicle only (control). The enterocytes (solid arrow) and GP cells (open arrow) are intact. X200

**Figure 5:**
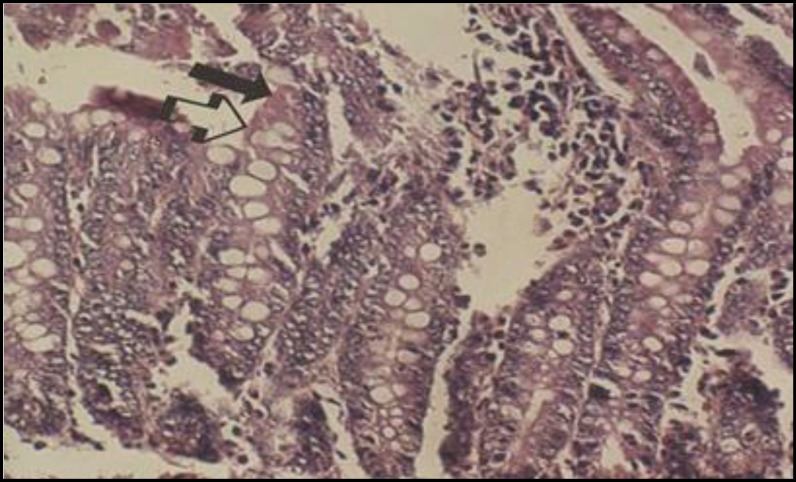
Light micrograph of rat small intestine treated with 600 mg/kg CAP. Showing extensive injury including loss of staining capacity, dilatation of the nucleus and cellular swelling in enterocytes (solid arrow )and GP cells (open arrow). H&E X 200

**Figure 6:**
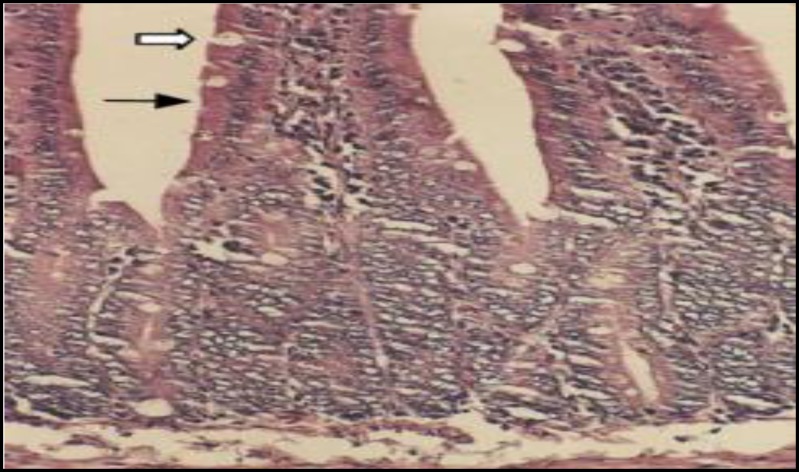
Light micrograph of rat small intestine pretreated with 80 mg/kg PB 30 min prior to administration of 600 mg/kg CAP. The enterocytes (solid arrow) and GP cells (open arrow) are intact. X200

In control rats, the liver tissue was intact with no obvious injury (Figure 1) while CAP could induce dose-dependent damage in hepatocytes (Figure 2). PB had no effect on hepatic cells, although it protected liver cell against CAP produced toxicity (Figure 3). The small intestine was intact in control rats (Figure 4). Muscularis mucosa did not show any significant findings and Serosal layer was intact without any inflammatory response. CAP could cause dose dependent injury in rat small intestine epithelial cells being predominant in rat enterocytes and goblet cells (Figure 5). PB had no effect on small intestine; nevertheless this agent protected the tissue against CAP-induced cell damage (Figure 6). 

## Discussion

Chloramphenicol is a broad-spectrum antibiotic that has high antimicrobial activity against a wide range of organisms including gram-positive and gram-negative bacteria, rickettsia, Chlamydia and mycoplasma. This antibiotic is particularly useful in infections caused by *Salmonella typhi* and *Haemophilus influenza *(1, 2 ). It is an effective and at the same time a potentially toxic antibiotic. A large body of evidence have indicated that this chemical induce liver toxicity (6-8). Although many drugs including antibiotics are prescribed orally, only few studies have been reported effects of this medicine on gastrointestinal epithelial cells (16, 17). 

We showed that CAP induced dose-dependent damage in liver and small intestine epithelial cells. Our histopathological observations indicated that there were dose-dependent degenerative changes of hepatic parenchymal cells. This was confirmed by induction of serum activity of AST and ALT. The increased level of these enzymes was similar to other investigators (8). Present study revealed that in control rats, there was no obvious injury in small intestinal tissues. Whereas mild to moderate inflammatory response were seen in mucosal and submucosal layers of rats small intestines treated with CAP. Result of CAP producing damage in enterocytes suggests that similar to the liver, small intestinal epithelial cells are susceptible to CAP-induced toxicity.

It has been known that CAP is detoxified by hepatic cytochrome p450 enzyme system (6). The presence of cytochrome p450 is reported in rat and human small intestine (11-14). Halper *et al* (1983) reported that CAP caused dose–dependent inactivation of hepatic cytochrome p450 (18). The mechanism by which CAP caused adverse effects on small intestine is not clear. However, inactivation of cytochrome p450 enzyme by CAP may be responsible for its adverse effects on small intestine.

The induction of hepatic cytochrome p450 activity leads to accelerated metabolism of many xenobiotics (19). Enhancements of CAP metabolism by Phenobarbital (PB) in rat liver were reported (15, 19). We found that hepatotoxicity and small intestine toxicity of CAP markedly reduced in PB-pretreated rats when compared to non-pretreated animals. 

Results of our study demonstrated a marked drug interaction between a sedative and an antibiotic. Induction of cytochrome p450 by PB may have increased glucuronidation or hydroxylation of the antibiotic and therefore increased CAP excretion.

Our observations along with others suggest that cytochrome p 450 is responsible for CAP detoxification. The finding that the extent of injury was more pronounced in 600 mg/kg CAP when compared to 400 and/or 200 mg/kg in treated rats further supports this hypothesis and indicates that destruction of cytochrome p450 more obvious occurs in rats following administration of higher dose. 

Pretreatment of rats with PB markedly decreased toxicity of CAP which provides support to the initiative that inadequate cytocrome p450 in small intestine is responsible for drug toxicity. It has been documented that PB enhances cytochrome p 450 activity in small intestine (14) which is in consistent with our observation and confirms that small intestinal epithelial cells have capacity to metabolize CAP.

## Conclusion

CAP caused biochemical and structural alterations in rat liver. Histopathological changes were also noted in small intestine epithelial cells. These finding suggest that similar to the liver, epithelial cells of small intestine are susceptible to CAP- induced toxicity. Amelioration of CAP induced toxicity by PB verifies that biotransformation of the antibiotic by cytochrome p450 is responsible for CAP detoxification. 
